# No gender-related differences in the severity of nephropathia epidemica, Germany

**DOI:** 10.1186/1471-2334-13-457

**Published:** 2013-10-03

**Authors:** Ellen Krautkrämer, Stephan Grouls, Eva Urban, Paul Schnitzler, Martin Zeier

**Affiliations:** 1 Department of Nephrology, University of Heidelberg, Im Neuenheimer Feld 162, 69120 Heidelberg, Germany; 2 Department of Virology, University of Heidelberg, Heidelberg, Germany

**Keywords:** Hantavirus, Puumala virus, Gender, Severity, Transmission

## Abstract

**Background:**

The number of cases of hantavirus disease caused by Puumala virus is increasing enormously in Germany within the last years. Men are overrepresented in hantavirus disease and differences in course and symptoms in relation to gender were reported from several countries. This study was conducted to define possible gender-specific risk factors and aspects of severity in hantavirus infections occurring in Germany.

**Methods:**

Characteristics, clinical parameters and symptoms were recorded in a retrospective analysis of 108 patients with serologically confirmed hantavirus infection treated in our department. This cohort corresponds in regard to age, time of infection and gender ratio to the characteristics of the overall cases reported in Germany.

**Results:**

The frequency of characteristic symptoms of hantavirus disease did not differ between males and females. The median of nadir and peak levels of clinical parameters did not exhibit relevant differences that would point to a more severe course in males or females. The clinical course and duration of hospitalization were similar for both sexes. No relevant differences in renal and pulmonary findings were observed. Males with hantavirus disease exhibited more cardiac findings than females.

To compare the unequal gender distribution of the rodent-borne Puumala hantavirus disease with the gender ratio of other infectious diseases, we analyzed the gender ratio for notifiable infections according to their mode of transmission. Our data revealed a general overrepresentation of men in infections carried by arthropods and rodents.

**Conclusions:**

In contrast to reports from other countries, no crucial differences in the symptoms, course or severity of hantavirus disease between infected men and female were observed in our cohort. However behavioural differences may account for the fact that men are more often affected by certain infectious diseases than females.

## Background

The epidemiology of zoonotic diseases is complex due to the multifaceted factors influencing occurrence of host species and thereby the risk of exposure to humans [1]. Besides these factors that have an impact on the transmission rate, infectious diseases often exert a broad spectrum of clinic severity. Hantavirus infection causes disease whose clinical picture varies in humans. The infection with hantaviruses in the Old World causes hemorrhagic fever with renal syndrome (HFRS). The mortality rate ranges from < 1% for Nephropathia epidemica (NE) caused by Puumala virus to 12% for infections with Hantaan and Dobrava-Belgrade virus [2]. The infection is characterized by sudden onset with fever, thrombocytopenia, hematuria, elevated levels of serum creatinine, and decreased serum albumin levels. The infection often results in acute renal failure with massive proteinuria.

Aside from virus-specific differences, the severity of symptoms depends on patient-specific characteristics. The high seroprevalence in comparison to the number of clinical cases indicates that infections are often undiagnosed because of mild or subclinical presentation. In Germany, the seroprevalence for hantavirus antibodies is 1–2% [3] and the average number of annually recorded hantavirus cases with an incidence of 0.87/100000 (range 0.09/100000–3.45/100000) persons from 2001 through 2012 [4].

Factors that have been described to be associated with disease severity include genetic predisposition and differences in immune response [5,6]. Gender-specific differences in symptoms, clinical parameters and mortality rates were identified for NE in Sweden and Finland and for HFRS in China [7-10]. Therefore, we analyzed the epidemiology and the clinical course of NE cases in Germany with regard to gender-related differences.

## Methods

### Study population

Hantavirus-infected patients (82 men and 26 women) hospitalized in the Department of Nephrology, University of Heidelberg, Germany from 2001 to 2012, were included. Heidelberg is located in Baden-Württemberg. The endemic area where the majority (56%) of reported hantavirus infections in Germany occur [4]. The diagnosis was confirmed by detection of circulating anti-hantavirus IgG- and IgM-antibodies. This study was approved by the Ethics Committee of the University Hospital of Heidelberg, Germany, and it adhered to the Declaration of Helsinki.

### Data collection

To analyze the epidemiology of infectious diseases, we used data surveyed by the Robert Koch Institute (RKI, Berlin, Germany). All confirmed hantavirus cases in Germany are reported to the RKI and are available at http://www3.rki.de/SurvStat. Clinical data were collected through a retrospective review of medical charts of the Department of Nephrology. Demographic data, underlying diseases, cardiac, ultrasound and laboratory findings were recorded. Nadir or peak levels of clinical parameters were expressed as median and range (min-max).

### Statistical analysis

Data were analyzed using GraphPad Prism 5.0. Normal distribution was tested by Kolmogorov-Smirnoff test. Clinical parameters of two groups were compared using Mann Whitney test or Student’s *t* test. Categorical variables were compared using Fisher’s exact test. p-values of < 0.05 were considered significant.

## Results

### Epidemiology

**Figure 1:**
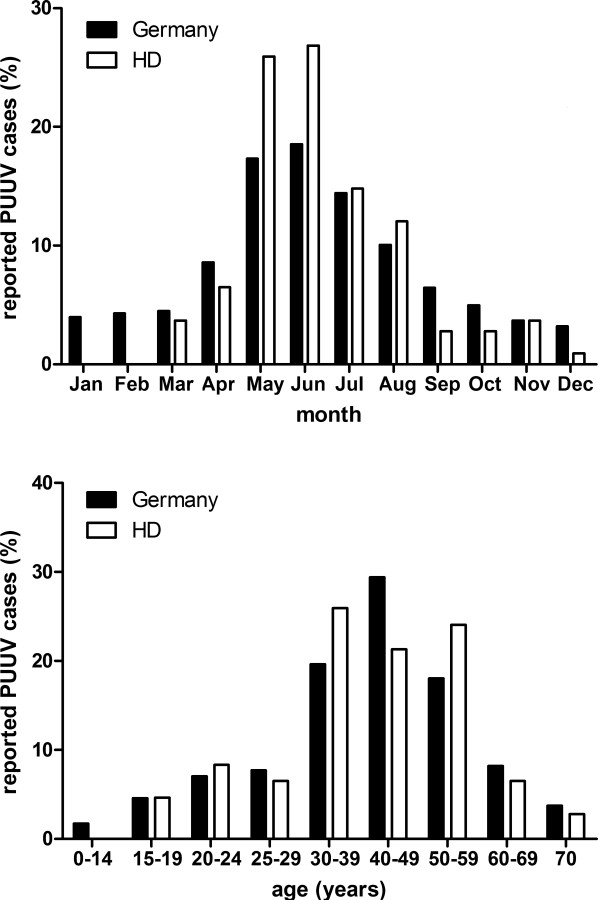
Seasonality of hantavirus Puumala illnesses and age distribution of our Heidelberg (HD) cohort and of patients with NE in Germany, RKI (2001–2012).

The survey of the RKI accounted for total of 7476 cases of infection with Puumala virus between 2001 and 2012 in Germany. The distribution of cases shows a pronounced peak between late spring and early summer. About 50% of cases occurred between May and July. About 30% of the infections affected persons in the age group of 40-to-49-years old (Figure 1). Hantavirus infection was diagnosed more often in men than in women. The male to female ratio was 2.53 between 2001 and 2012 and ranged from 1.85 in 2008 to 3.41 in 2002.

To analyse gender-specific differences of hantavirus infection, we analyzed data of 108 (82 males and 26 females) patients treated in the Department of Nephrology, University of Heidelberg, Baden-Württemberg, Germany. Most patients were treated between May and July. The patients median age was 42 years (18–81 years) and the male to female ratio was 3.15 (Figure 1). In summary, the epidemiological parameters of our cohort correspond to the characteristics of the overall reported cases in Germany.

### Clinical and laboratory findings

Men and women of our cohort were similar in age, in the time they spent with symptoms before hospital admission and in hospital length of stay. Symptoms on admission were flu-like, with headache, fever, nausea, side/back and abdominal pain. Additional symptoms were night sweat, diarrhea and ophthalmological disorders (Table 1). The frequency of most symptoms did not differ between men and women. Myalgia and edema were more often present in female patients. The laboratory examinations (Table 2) displayed elevated leukocyte counts and elevated levels of serum creatinine, lactate dehydrogenase (LDH) and C-reactive protein (CRP) in both sexes. Platelet numbers and levels of serum albumin were decreased. The median minimal level of serum albumin was lower in female patients than in male patients. Renal findings comprised oliguria/anuria, proteinuria and hematuria. Hemodialysis treatment was required for five patients and was not more frequently used to treat male or female patients (Table 3). Pulmonary involvement presenting as pleural effusion and lung infiltration were observed on both sexes. However, most patients with cardiac findings in ECG were male (Table 4). In summary, no gender-related differences in the clinical signs, maximum or minimum levels of laboratory parameters, renal or pulmonary findings that would be relevant for the clinical outcome were observed.

**Table 1:** Characteristics and symptoms of 108 patients with laboratory-confirmed Nephropathia epidemica

	**Male**	**Female**	**P value**
	***n*** **= 82**	***n*** **= 26**	
Median age in years (range)	42 (18–81)	40.5 (19–68)	0.4083
Median admission after onset of symptoms in days (range)	6 (0–17)	5 (3–17)	0.9607
Median duration of hospitalization in days (range)	7 (0–29)	7 (1–12)	0.5269
Fever	70 (85.37%)	22 (84.62%)	1.0000
Headache	60 (73.17%)	20 (76.92%)	0.8014
Nausea	51 (62.20%)	21 (80.77%)	0.0975
Side/back pain	49 (59.76%)	21 (80.77%)	0.0611
Abdominal pain	46 (56.10%)	14 (53.85%)	1.0000
Night sweat	25 (30.49%)	4 (15.38%)	0.2032
Diarrhea	25 (30.49%)	8 (30.77%)	1.0000
Ophthalmological symptoms	21 (25.61%)	10 (38.46%)	0.2218
Myalgia	20 (24.39%)	15 (57.69%)	0.0033
Weight loss	18 (21.95%)	4 (15.38%)	0.5833
Cough	15 (18.29%)	4 (15.38%)	1.0000
Edema	14 (17.07%)	10 (38.46%)	0.0308
Dyspnea	7 (8.54%)	6 (23.08%)	0.0777

**Table 2:** Laboratory findings in patients with NE

	**Male**	**Female**	**P value**
	***n*** **= 82**	***n*** **= 26**	
	**Median (range)**	**Median (range)**	
Serum creatinine max (mg/dl)	5.61 (1.4–14)	5.69 (1.3–10.89)	0.9484
Leukocytes max (G/L)	11.45 (5.66–26.6)	12.77 (7.2–19.34)	0.5017
Platelets min (G/L)	88.5 (24–475)	91.5 (32–353)	0.9685
Serum albumin min (g/L)	33.3 (21.4–45.2)	30.4 (24–37.6)	0.0027
CRP max (mg/L)	57.35 (3.4–208.8)	55.65 (24–144.3)	0.6559
LDH max (U/L)	364.5 (217–918)	358 (228–1032)	0.9828

**Table 3:** Frequency of renal findings among patients with NE

**Renal findings**	**Male**	**Female**	**P value**
	***n*** **= 82**	***n*** **= 26**	
Oliguria/anuria	40 (48.78%)	15 (57.69%)	0.5026
Proteinuria	72 (87.80%)	25 (96.15%)	0.2906
Hematuria	49 (59.76%)	19 (73.08%)	1.0000
Dysuria	11 (13.41%)	0 (0.00%)	0.0626
Nycturia	20 (24.39%)	5 (19.23%)	0.7903
Dialysis	3 (3.66%)	2 (7.69%)	0.5920

**Table 4:** Frequency of cardiac and pulmonary findings among patients with NE

	**Male**	**Female**	**P value**
	***n*** **= 73**	***n*** **= 25**	
Patients with cardiac findings in ECG	47 (64.38%)	8 (32.00%)	0.0200
	**Male**	**Female**	**P value**
	***n*** **= 67**	***n*** **= 22**	
Patients with pulmonary findings in the x-ray	28 (41.79%)	9 (40.91%)	0.1971

### Clinical courses

**Figure 2:**
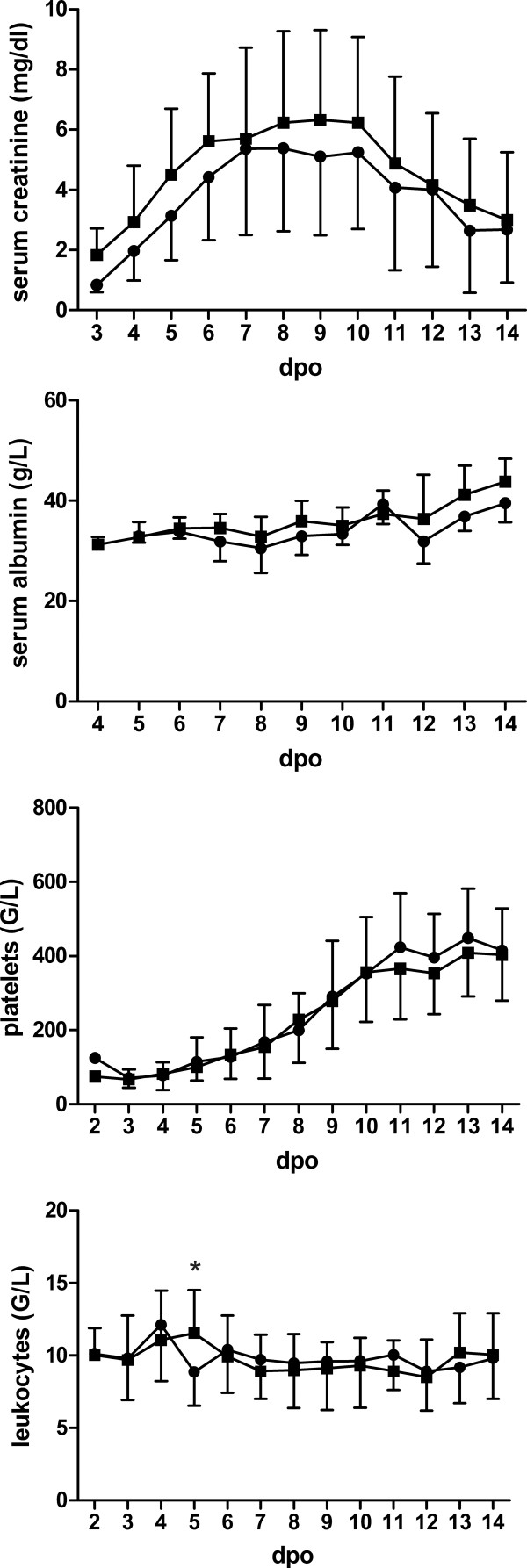
**Course of laboratory parameters of 22 male and 21 female patients with NE, Heidelberg.** Shown is the mean and standard deviation. Squares: males; circles: females. dpo: days post onset. *P < 0.05, Student’s *t* test.

To evaluate the clinical course of infection in men and women, we analyzed laboratory parameters over time (Figure 2). The mean levels of serum creatinine, leukocytes, platelets and CRP do not vary between men and women during the 14 day period of observation. Peak and nadir levels of both sexes were observed at the same time point after onset of symptoms and duration of hospitalization does not differ between men and women.

### Cause of infection

Men are more frequently affected by hantavirus disease than women. Therefore, we evaluated the activities associated with hantavirus infection. The percentage of women that were exposed during free-time is higher than the percentage of men (84.21% vs 53.85%). In contrast, men are more often infected during occupational activities than women (21.54% vs. 5.26%). Unfortunately, 24.62% of men and 10.53% of women were not sure of the mode of infection. Studies in larger cohorts will be necessary to confirm these observations.

**Figure 3:**
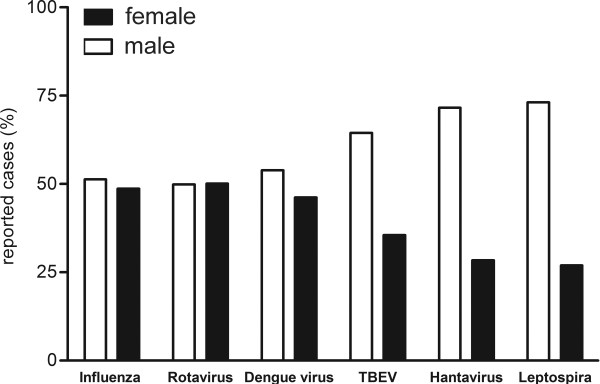
Gender distribution of patients with infectious diseases, RKI, Germany, 2001–2012.

To compare the gender distribution of different infectious diseases, we analyzed the gender ratio of several notifiable infectious diseases in Germany (Figure 3). The analysis revealed that the gender distribution depends on the mode of the transmission of the disease. Infections transmitted via contaminated objects or from men-to-men display an almost equal gender ratio. In contrast, infections transmitted via arthropods or rodents affect men more often than women. These results indicate that differences in occupational and free-time activities that are associated with a higher risk for exposition may account for the unequal gender ratio.

## Discussion

The underlying mechanisms for the variety of severity of infectious diseases are still not completely understood. For hantavirus infections, several patient-specific characteristics such as specific alleles for HLA and TNF-α that are associated with the clinical courses have been identified [5,6,11-15]. Numerous gender-specific differences in hantavirus disease were also often discussed. The most striking difference is the higher incidence of hantavirus disease in males that is observed in countries with hemorrhagic fever with renal syndrome [16] as well as in New World countries with hantaviral cardiopulmonary syndrome (HCPS) [17]. Several other sex-dependent differences were reported that may influence the outcome of the disease. Studies in Scandinavian patients suffering from infection with Puumala virus analyzed cytokine response and expression of the estrogen receptor [7,8]. Furthermore, a higher risk for young male patients for severe central nervous system complications during infection with Puumala virus was described [9]. Interestingly, despite higher incidence in men a higher fatality rate for women was observed in patients with HFRS in China and with NE in Sweden [10,18]. Underlying gender-dependent differences in the clinical course of hantavirus disease leading to fatal outcome could not be identified so far. As reported for hantavirus disease in other countries, the imbalanced distribution of cases is also observed in Germany and in the cohort that was treated in our department. However, symptoms, laboratory parameters and clinical course do not differ enormously between men and women in our patient cohort. Solely, the median level of minimum serum albumin was lower in female than in male patients. Renal findings such as oliguria/anuria, proteinuria and hematuria as well as the requirement of dialysis were more often observed in women. However, these observations do not reach statistical relevance. The imbalanced male to female ratio of hantavirus disease is often explained by the higher risk of exposition for men. Analysis of the M:F ratio for notifiable infectious diseases in Germany reveals a strong relationship between the mode of transmission and the number of infections in men and women. These findings indicate that men are more often affected by vector-borne diseases due to a higher expositional risk. Interestingly, seroprevalence in forestry workers of a non-endemic area in Germany shows no difference between men and women [19]. This would support our observation that differences in behavioral factors such as occupational and leisure activities account for the unequal gender distribution and not a higher susceptibility of men for arthropod-borne and an even higher for rodent-borne diseases. In contrast, equal seroprevalences for males and females were observed in randomly selected individuals in endemic areas in Sweden and Germany [3,20]. This would lead to the conclusion that hantavirus infection in women is subclinical or less severe. Therefore, general gender-related differences in the susceptibility, severity or course of hantavirus infection could not be identified so far. Studies differ in hantavirus species and geographic area. Additional limitations are often the small size of cohorts with few women and the lack of information about the mode of transmission. The characteristics of host reservoir populations are also important for the analysis of risk factors for human infection. Further studies are necessary to define the relationship between gender and risk factors for severe courses of hantavirus disease caused by Old World hantaviruses Hantaan, Puumala and Dobrava-Belgrade virus as well as for HCPS-causing New World hantavirus species.

## Conclusions

Gender-related differences in infectious diseases were often discussed. For hantavirus infection caused by diverse species several differences in regard to severe complications and clinical parameters were described. However, the sole difference that we could observe in our cohort is the unequal distribution of Puumala virus induced hantavirus disease between men and women. Differences in the symptoms or severity of hantavirus disease were not present between the male and female group. We observed a strong association between gender ratio and mode of transmission of infectious diseases. Further epidemiological studies on larger cohorts will be necessary to clarify the role of the mode of transmission in gender-specific differences of vector-borne diseases.

## Competing interests

The authors declare that they have no competing interests.

## Authors’ contributions

EK, SG designed the study and drafted the manuscript. PS, EU performed the data acquisition. MZ participated in the design of the study and helped to draft the manuscript. All authors read and approved the final manuscript.

## Pre-publication history

The pre-publication history for this paper can be accessed here:

http://www.biomedcentral.com/1471-2334/13/457/prepub
